# Research on patient-centered design for post-stroke depression patients based on SEM and comprehensive evaluation

**DOI:** 10.3389/fpubh.2023.1120596

**Published:** 2023-05-26

**Authors:** Yuxuan Li, Qi Zhang, Xing Fang

**Affiliations:** ^1^Department of Information Design, School of Design, Wuhan University of Technology, Hubei, China; ^2^Department for Public Health, Wuhan Jinyintan Hospital, Wuhan, Hubei, China

**Keywords:** virtual reality therapeutic landscape, patient-centered design, multimodal interaction, structural equation model, comprehensive evaluation

## Abstract

**Introduction:**

Since COVID-19, medical resources have been tight, making it inconvenient to go offline for the sequelae of diseases such as post-stroke depression (PSD) that require long-term follow-up. As a new digital therapy, VRTL began to gain popularity.

**Method:**

The research is divided into two parts: pre-test and post-test. In the pre-test, an evaluation method integrating reality-based interaction (RBI), structural equation model (SEM), analytic hierarchy process (AHP), and entropy weight method is proposed. In the post-test the patients’ physiological indicators (Diastolic blood pressure, systolic blood pressure and heart rate) are measured to verify the effectiveness of RBI-SEM model using *T*-test method.

**Results:**

In the pre-test, using SEM, it was confirmed that *P_i_* physical awareness, *B_i_* body awareness, *E_i_* environmental awareness, and *S_i_* social awareness were significantly correlated and positively affected VRTL satisfaction (*p* >> F 0.217; *B* >> *F* 0.130; *E* >> *F* 0.243; *S* >> *F* 0.122). The comprehensive weight ranking based on RBI-SEM considered light environment (0.665), vegetation diversity (0.667), accessible roaming space (0.550) et al. relatively of importance. And *T*-tset in the post-test experiment considered that the data of the two measurements before and after the VRTL experience, systolic blood pressure (*p* < 0.01), diastolic blood pressure (*p* < 0.01), and blood pressure (*p* < 0.01) were significantly decreased; one-way ANOVA concluded that there was no significant difference in the changes of blood pressure and heart rate among participants of different ages and genders (*p* > 0.01).

**Conclusion:**

This research validated the effectiveness of RBI theory for VRTL design guidelines, established an RBI-SEM based VRTL evaluation model, and the output VRTL for PSD in the older adults was confirmed to have significant therapeutic benefits. This lays the foundation for designers to decompose design tasks and integrate VRTL into traditional clinical treatment systems.

**Contribution from the public or patients:**

Four public health department employees helped to improve the research’s content.

## 1. Introduction

Rehabilitative Landscape (TL) combines a specific natural environment, building environment, social conditions, and human perception to construct an atmosphere conducive to rehabilitation. TL is composed of Biophilia Hypothesis (BH) (1), and it has been proven through comparative experiments that the rehabilitation landscape can accelerate the recovery after the operation and reduce the use of drugs. In addition, TL plays an outstanding role in the treatment of psychological diseases. According to Psychologist Kaplan’s research, the natural landscape environment has a significant positive effect on the overall health of older adults, especially the enhancement of psychological satisfaction and pleasure, which can effectively combat psychological problems like depression (2).

In 2001, Marcus defined the outdoor environment as an “action space” in his research, analyzed the successful cases of rehabilitation environments in geriatric sanatoriums and hospitals, discussed the general layout of outdoor landscape environment design in geriatric residential areas, emphasized the importance of landscape rehabilitation function to the older adult residents, and proposed suggestions for the design of rehabilitation environment for the older adults (3). In addition, Zeisel et al. also covered numerous features of the rehabilitation landscape, and how they relate to Alzheimer’s disease and other mental diseases, including reducing physical and mental discomfort, improving quality of life, maintaining physical function, delaying aging, and extending residual cognitive ability (4).

The application of VR in medical industry began with the construction of simulated organs for surgical training. Rothbaum et al. (5). proposed Virtual Reality Therapy (VRT) to apply VR to the rehabilitation treatment of psychological diseases. At present, virtual exposure therapy (VRET) is widely used in VRT (6). and can effectively treat post-traumatic stress disorder (PTSD) by controlling and reproducing virtual exposure sources compared with live exposure. Combining VR technology with modern rehabilitation medicine, VRT shows advantages in many rehabilitation fields.

Virtual Reality Rehabilitation Landscape (VRTL) is an extended treatment approach to VRT (7). By simulating an immersive 3D virtual surroundings, characters and landscapes, patients can exercise in an immersive landscape to achieve therapeutic benefits (8). Through the VRTL for Parkinson’s disease patients, Klinger (7) found that if the patients were given a clear direction of action and an interactive landscape, rehabilitation training with VR could reduce the time users completed their actions in reality. Chandler (8) measured the heart rate and blood pressure of participants in various combinations of games and exercises, and the experiment demonstrated that there were no significant differences in physiological parameters between the VRTL and physical exercise groups. It is proven that VRTL has an exercise effect and can promote health. Similarly, with the maturity of technology, VRTL has been applied to more and more fields of disease rehabilitation, including improving the cognitive level of stroke patients (9), treating Parkinson’s (10), overcoming psychological trauma (11), and rehabilitating limbs and balanced perception (12).

Robert J put forward reality-based interaction (RBI), pointing out that the interaction of the virtual world is the simulation of the objective world (13). At the beginning of the theory, RBI was mainly used as an evaluation tool to improve the user interface (UI) for the VR environment. Adamides et al. applied RBI to the design of a remote control system for agricultural machinery (14). Similarly In order to demonstrate the viability of RBI as a design guiding tool, Tairan Li et al. (15) proposed the design theory of the space-three-dimensional object-menu-interactive approach (SOMM) in VR. They then utilized it in industrial assembly training. Researchers have discovered that RBI is applicable to other design processes besides UI design since RBI is being used in more and more design practices. Using four criteria based on RBI, Audrey et al. (16) further interpreted user interaction behavior: *P_i_* physical awareness, *B_i_* body awareness, *E_i_* environmental awareness, and *S_i_* social awareness. *P_i_* denotes the basic spatial components and sensory indicators in VRTL; *B_i_* denotes the interactive functions and actions that can be realized in VRTL; *E_i_* denotes the landscape element in VRTL and is a concrete representation of landscape healing, similar to, e.g., Li et al. (17). Using sound, location, and weather as measured variables of the environment in their study, combining them with daily activities and physiological information, it was concluded that they could respond to changes in the self-perception of the subject through long-term recording; *S_i_* denotes the achievable social content and emotional elements in VRTL (18), and it is worth noting that the sense of direction is greatly diminished in immersive VR, thus guiding elements such as haptics are may necessary (19). Additionally, some scholars have summarized the theoretical framework of interaction methods from special technology or environment (20). They proposed theoretical models based on qualitative data, such as mind-flow based interaction, gesture recognition oriented interaction, and multi-angle interaction.

The above research shows that the application of RBI and VRTL in rehabilitation medicine is expanding continuously. However, there is a dearth of research on how to construct VRTL. So, the VRTL design at this point lacks examination and analysis of patients’ demands and is primarily focused on conventional therapies or designed solely based on the developer’s subjective experience (18, 20). Therefore, it is urgent to construct a VRTL design process centered on patient experience and explore a reasonable method for evaluating design elements. In traditional user needs analysis, the subjective evaluation method hierarchy analysis (AHP) is widely used, which can effectively reflect expert opinions and quantify the indicators. Constant and entropic weighting is used in combination due to an overreliance on objective weighting methods such as subjective opinions (21). The structural equation model (SEM),widely used in the field of user experience, comprehensively evaluates the constructed model and verifies its rationality by using various statistical methods (21). Unlike traditional evaluation methods, SEM can estimate the relationship between multiple potential variables, retain and remove measurable variables with weak correlation, and is suitable for VRTL design based on RBI theory.

In general, this paper presents two research questions. Firstly, the RBI-SEM model is proposed, hypothesizing that *P_i_* physical awareness, *B_i_* body awareness, *E_i_* environmental awareness, and *S_i_* social awareness are significant correlation to VRTL, and patient evaluation scales are collected through VRTL experiment to verify the theoretical. In addition, the RBI-SEM path coefficients were integrated with AHP and entropy weight method to rank the design indicators (later referred to as pre-test). Secondly, physiological indicators (including heart rate, systolic blood pressure, and diastolic blood pressure, later referred to as post-test) were collected from patients before and after the VRTL experience experiment to verify the therapeutic effectiveness of VRTL on PSD, to provide empirical research support for the design conception, and to further validate the feasibility of the design methodology.

## 2. Methodology and research processes

### 2.1. Research processes

**Figure 1:**
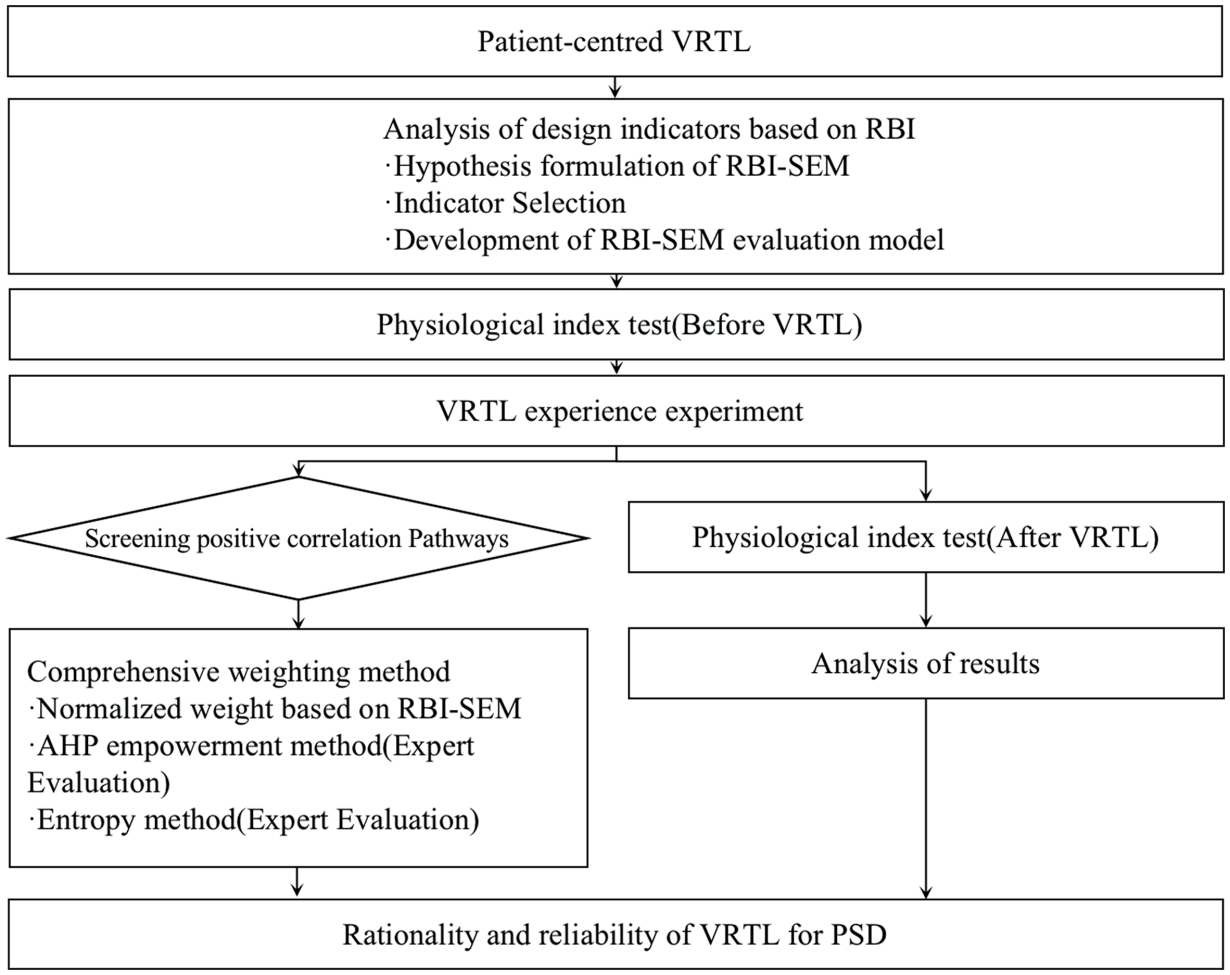
Research process flowchart.

Traditional weighting methods are based on the subjective experience of researchers and have limitations. Therefore, this study precedes the assumptions made by SEM, which is widely used in multithreaded planning, to validate the validity of the model by collecting patient experience data according to the test software. After evaluating and calculating each algorithm based on RBI-SEM, the weights are linearly combined and sorted to guide the design practice. Specific steps are as follows in Figure 1. There are three main steps to the research process:

Step 1: Assumptions proposed and RBI-SEM model established. SEM model is proposed based on RBI theory and all potential variables are assumed to contribute positively to VRTL design; In the case study, VRTL test software is run to collect evaluation data of each measurable variable from patients to confirm the model’s validity.
Step 2: Comprehensive evaluation model for VRTL. Based on the SEM path coefficient, the design index is comprehensively evaluated by linear synthesis using the normalization algorithm, subjective weighting method AHP and objective weighting method Entropy Weighting method.
Step 3: Measurement of physiological indicators. After the patients have experienced the VRTL, the validity of VRTL design is verified by measuring physiological indicators such as blood pressure and heart rate, and compared with the effect of traditional rehabilitation therapy.

### 2.2. Introduction to the scale used

During the process of this research, it was necessary to collect patient or expert opinions through various scales multiple times. Questionnaires were needed in Pre-test for screening PSD patients, screening VRTL evaluation indicators, and evaluating the VRTL experience. Accordingly, a 7-point Likert scale was used throughout the study, with higher values representing greater satisfaction or agreement. The details are as follows:

1. BE-PSD scale: This scale was used to screen patients with PSD from stroke patients to experience VRTL. Based on the results of Takeuchi et al. (22), the present research rated the symptoms from mild to severe with a mean value >2.2 on several dimensions such as mental status, thought disorder, and negative condition to determine poor mental well-being. Additionally, Everton et al. (23) found that complications such as vomiting caused by stroke are also likely to cause depression, so physical condition was added to the scale as an additional evaluation dimension.
2. RBI-SEM indicator screening scale: This scale was used to screen indicators that comprise the theoretical model from a larger number of VRTL indicators. Structural equation modeling was used in the study to argue whether *P_i_*, *B_i_*, *E_i_*, and *S_i_* in RBI theory were significantly correlated with VRTL, while more theoretical approaches emerged in the literature studies, which were screened by expert scoring.
3. RBI-PRS scale: Perceived Restorativeness Scale (PRS) was proposed by Hartig et al. (24)to evaluate the restorativeness of the environment from being away, fascination, cooperation, compatibility, four dimensions. This scale has been widely used in landscape restoration evaluation (25, 26). The questionnaire is the most important information collection tool for RBI-SEM, and the scale was used to collect evaluation information after VRTL experience to provide data for the validation of RBI-SEM model and index evaluation. Also, The scores of negative questions were being revised for consistency.

## 3. Theoretical modeling and analysis

### 3.1. Indicator screening

In VRTL, the software simulates how patients view, analyze, and engage with landscapes, mostly using their five senses. Patients also receive treatment using these procedures. Various factors will affect the final cognitive outcomes during the recognition process. Generally, there are two types of influencing factors: subjective influences (from the cognitive subject) and objective factors (from the cognitive object). Different types of users have different needs for the rehabilitation landscape, and there are also variances in the performance of restrictive factors of different geographical characteristics. Therefore, it is necessary to specify a set of elements evaluation index system and establish the weights of each index factor. Six fundamental principles—scientific, methodical, integrated, purposeful, practical, and operable—will be closely followed in the establishment of the hierarchical structure of the virtual rehabilitation landscape evaluation. The reliability and validity of the evaluation outcomes are, in part, determined by these principles, which also play a guiding role in developing an acceptable evaluation index system and a scientific evaluation model.

Based on the above literature and related research, the optional VRTL design methods are concluded for selection by professionals through RBI theory and existing VR technology capabilities (12). In formula (1), 15 relevant experts advise that H is the correlation of this index to VRTL design, G is the number of experts who choose this index, *N* is the number of valid questionnaires received, and *H* is <0.5, then this index is not suitable for VRTL design. As shown in Table 1, it summarized and classify the common and major influencing factors in VRTL for older adults with depressive tendencies. According to RBI, the elements of evaluation are classified as physical perception, action perception, environmental perception and social perception. According to the above four categories, 13 rehabilitation elements related to research concepts are selected as measurable variables.


(1) $$H=\frac{G}{N}$$

**Table 1:** Indicator selection for RBI-SEM.

Structural latent variable	Measurable variable	Design elements	*H_i_*	Judgement
*P_i_* Phyics awareness	P1	Light environment	0.534	Retain
	P2	Acoustic environment	0.600	Retain
	P3	Color suitability	0.600	Retain
	P4	Tactile perception	0.534	Retain
	P5	Olfactory perception	0.200	Delete
*B_i_* Body awareness	B1	Interactive landscape	0.867	Retain
	B2	Motion perception	0.268	Delete
	B3	Boundary space	0.534	Retain
	B4	Barrier-free design	0.133	Delete
	B5	Travel speed	0.733	Retain
	B6	Spatial scale	0.200	Delete
*E_i_* Environmental awareness	E1	Scene scale	0.600	Retain
	E2	Spatial performance	0.067	Delete
	E3	Scene artistry	0.867	Retain
	E4	Vegetation diversity	0.667	Retain
	E5	Slope landscape	0.133	Delete
	E6	Biological landscape	0.067	Delete
	E7	Water landscape	0.534	Retain
*S_i_* Social awareness	S1	Recall element	0.733	Retain
	S2	Identification system	0.534	Retain
	S3	Roaming Space	0.867	Retain
	S4	Service elements	0.867	Retain

### 3.2. Theoretical model construction

**Figure 2:**
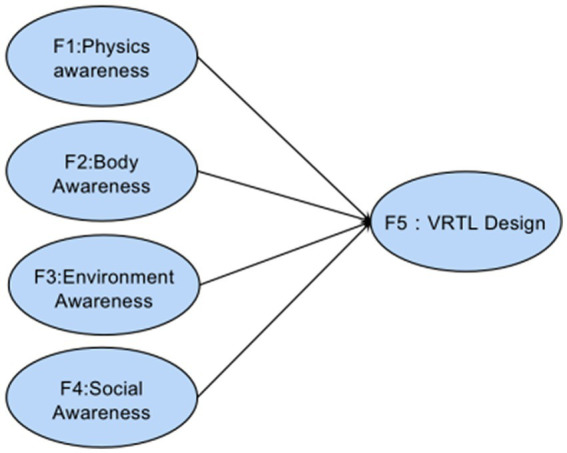
Basic model.

**Figure 3:**
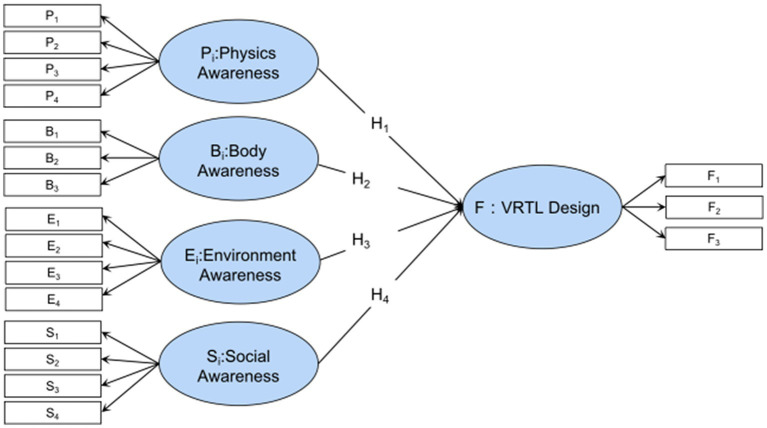
RBI-SEM theoretical model.

Traditional factor analysis struggles with models that have complex subordination relationships, such as one index subordinating to multiple factors or considering higher-order factors, but structural equation analysis allows for more complex models. Traditional path analysis only estimates each path’s strengths (relationship between variables). In structural equation analysis, different models can be calculated to fit the same sample data as a whole to determine which model is closest to the relationship presented by the data, in addition to the above parameter estimates. Meanwhile, the design elements of VRTL are interrelated according to RBI design theory, so the rationality of the index system corresponding to each potential variable cannot be analyzed and the weight coefficient can be determined separately. Hence, the basic model in Figure 2 is built on the four dimensions of RBI theory. The RBI-SEM model was constructed from the measurable variables in Table 1, as shown in Figure 3, assuming that the four RBI-based latent variables *P, B, E, S* are significantly correlated with and providing positive effects on VRTL design satisfaction.

### 3.3. Model calculation method

#### 3.3.1. Weighting algorithm based on the coefficient of action path

Before calculating the weight of SEM, a measurement model is as shown in Figure 3, and the relationship between the measurable variable and the latent variable is as follows. Where X denotes the external indicator; Y represents an endogenous indicator; _x stands for the embodiment of exogenous index X and exogenous potential variable *ξ* Relationship; _y is an endogenous indicator Y and an endogenous potential variable *η* Links; *δ*, *ε* denotes the Measurement errors for X and Y, respectively.

(2) $$X=\Lambda_{x}\xi +\delta$$

(3) $$Y=\Lambda_{y}\eta +\varepsilon$$

The path equation, which reflects the correlation between the underlying variables, is described as follows:

(4) $$n_{i}=\alpha +Bn_{i}+\psi x_{i}+\rho_{i}$$

where ni:p × 1 Vector of the number of observed endogenous variables. Xi:q × 1 Vector of the number of observed exogenous variables. *α*: *P* × 1 Vector of the intercept of the regression equation. *Β*: *P* × *P* Matrix of the slope of the regression equation. *P* × The matrix of the slope of the *Q* regression equation. *Ζ*: *P* × 1 Disturbance factor is the vector of residuals.

According to (2)-(4) the action path coefficient value (i.e., factor load, the larger the value, the greater the influence of the measurable variable on the latent variable. In the SEM model of VRTL, the degree to which the design element can provide user satisfaction) can be obtained. The normalization algorithm is used to get the index weight as follows:


(5) $$W_{s}=\varphi_{Xi}/\overset{n}{\underset{i=1}{\sum }}\varphi_{Xi}$$

Formula: physical perception, action perception, environmental perception and social perception are, respectively, the function path coefficients of the first indicator; *Wx_i_* is the first index weighting value *I* = 1, 2,…, 13 for physical perception, action perception, environmental perception and social perception, respectively.

#### 3.3.2. Comprehensive empowerment

The subjective weighting method AHP is widely used in various index evaluations. The common methods for calculating the weighting vector include the how-average method, arithmetic average method and feature vector method (27). Three methods are applied in this paper to calculate the index of conceptual design according to the assignment of experts. and the average value is taken as the final subjective weight to ensure the accuracy of the assignment. The detailed procedures are as follows:

Step 1: Assume that the judgment matrix is


(6) $$A_{\mathrm{ij}}=\left[\begin{array}{cccc} a_{11} & a_{12} & \cdots & a_{1n}\\ a_{21} & a_{22} & \cdots & a_{2n}\\ \vdots & \vdots & \vdots & \vdots \\ a_{n1} & a_{n2} & \cdots & a_{\mathrm{nn}} \end{array}\right]$$

Step 2: Obtain the subjective weight vector


(7) $$W_{1}=\frac{1}{n}\overset{n}{\underset{j=1}{\sum }}\frac{a_{ij}}{\overset{n}{\underset{k=1}{\sum }}a_{kj}},i=1,2,\cdots ,n$$

When constructing the judgment matrix, we rely on expert scores to get quantitative data. Because of the unilateral subjectivity of experts’ perceptions, it is difficult to ensure the existence of errors, which leads to contradictions in the information of the judgment matrix and affects credibility. Therefore, consistency testing of the constructed judgment matrix ensures the effectiveness of subjective decisions. Represent consistency metrics as CI , Where the maximum eigenvalue is represented as $\lambda_{\max}$ ， n stands for the order. In practical applications, a consistency check should be carried out before weight calculation. The specific formulas are as follows:


(8) $$CI=\frac{\lambda_{\max}-n}{n-1}$$

(9) $$CR=\frac{RI}{\mathrm{CI}}$$

AHP can effectively assign subjective weight to design indexes, but experts are prone to make misjudgments and misjudgments due to their mental burden when confronting high-order matrices. To solve the subjective misjudgment caused by experts, the objective evaluation method is introduced to correct the index elements, which can effectively reduce subjectivity in problem (27). The entropy method, as an objective evaluation method, gives the objective weight of index based on the information Entropy, and information Entropy $H_{j}$ represents the measurement of the uncertainty of random variables in a system, or the uncertain impact of indicators on the final results in the evaluation system (with uncertain weights). The smaller the indicator, the greater the difference between the representative and other indicators. The more information provided and the greater the weight. The specific calculation method is as follows:

Step 1: Construct the decision matrix and standardize it. Assuming there are m elements and *N* expert decision scoring, the evaluation decision matrix is:

(10) $$X_{ij}={\left[\begin{array}{cccc} x_{11} & x_{12} & \cdots & x_{1n}\\ x_{21} & x_{22} & \cdots & x_{2n}\\ \vdots & \vdots & \vdots & \vdots \\ x_{m1} & x_{m2} & \cdots & x_{mn} \end{array}\right]}_{m\times n}$$

Step 2: Calculate the normalized matrix and form the normalized matrix by the ratio of column vectors. The sum of all elements is in the normalized matrix Q . The calculation process is as follows:


(11) $$x_{ij}=\frac{x_{ij}-\min\left(x_{ij}\right)}{\max\left(x_{ij}\right)-\min\left(x_{ij}\right)},j=1,2,\cdots n$$

(12) $$Q={\left(q_{ij}\right)}_{m\times n},i=1,2,\cdots ,m,j=1,2,\cdots ,n$$

where $q_{ij}=x_{ij}/\overset{m}{\underset{i=1}{\sum }}x_{ij}^{2}$ Step 3: Calculate the information Entropy of the index based on the normalization matrix $Q={\left(q_{ij}\right)}_{m\times n}$ Information Entropy of Index **J** is obtained by using Formula (13) $H_{j}$ .


(13) $$H_{j}=-K\overset{n}{\underset{i=1}{\sum }}p_{ij}\cdot \ln\left(p_{ij}\right),j=1,2,\cdots ,n$$

where *K* is the adjustment coefficient, *K* = 1/lnn.

Step 4: Determine the objective weight of the index. According to the information Entropy, the weight of index J is obtained by formula (10).


(14) $$W_{2}=\frac{1-H_{j}}{m-\overset{m}{\underset{i=1}{\sum }}H_{j}},j=1,2,\cdots n$$

Step 5: RBI-SEM design method and subjective and objective weights are used to modify each other and get comprehensive weights of indicators. In this research, the weights based on SEM are equally important as subjective and objective weights. The formula for calculating the comprehensive weights is as follows:


(15) $$\omega_{j}=W_{s}+\frac{W_{1}+W_{2}+\cdots W_{n}}{n},n=1,2\cdots n$$

## 4. Design practice

### 4.1. Population and sample

Stroke is one of the commonly occurring acute cerebrovascular diseases in the older adults and, in addition to physical damage, it can have serious psychological and emotional consequences (21, 22). And the most frequently seen psychiatric problem is post-stroke depression (PSD). Centered on the characteristics of PSD patients, this study focuses on acute stroke patients who were hospitalized in the Department of Neurology at JinYintan Hospital, Wuhan City, Hubei Province, from March 2021 to August 2021. Data are collected the patients hospitalized for stroke for at least 3 days. If the patient has recently undergone surgery, the attending physician’s recommendation will be taken into account and the patient will be selected as having a relatively stable condition. All final samples satisfy the following conditions: age above 55; clear awareness; good reading ability, no cognitive and communication barriers; stroke diagnosed by a head CT or MRI. The basic information of the patient participants is shown in Table 2.

**Table 2:** Demographics.

Variable	Value	Frequency	% (rounded)
Sex	Male	47	53
	Female	41	47
Age	55–59	26	30
	60–64	33	38
	Over 65	29	32
BE-PSD Score	1–2.2	32	36
	2.2–6	56	64
Familiarity with VR	Never use	43	49
	Rarely use	35	40
	Sometimes use	7	8
	Often use	3	3

### 4.2. Pre-test task definition

**Figure 4:**
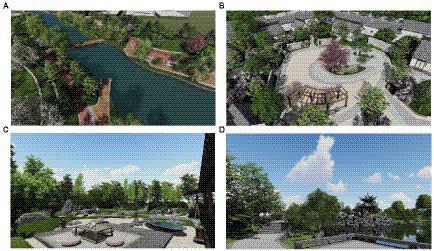
**(A)** Preview of the overall environment with mainly natural landscape (the first minute). **(B)** Preview of the overall environment with mainly humanistic architecture (the first minute). **(C)** Interactive landscape, drinking tea. **(D)** Roaming space and sculpture and other landscape displays.

The purpose of Pre-test is to evaluate the importance of each design element in VRTL to construct and validate the RBI-SEM evaluation model for Post-test. The Oculus Rift + Touch system was used as the interaction hardware in the test procedure, and the test screen of the VRTL is shown in Figure 4. In this highly immersive VRTL testing procedure, patients can switch between motion and first person perspective.

After a 5-min VRTL experience (1 min overall preview, 4 min self-guided tour). A total of 88 patients participated in the experiment, and after a 5-min VRTL experience (1-min overall preview and 4-min self-guided tour), patients evaluated the importance of the design indicators in order according to the four dimensions in the RBI-PRS scale (each indicator consisted of four dimensional scores, and the mean was taken as the final assessment). Considering the complexity of VRTL evaluation, 10 graduate medical students and 5 university faculty members were invited to be responsible for AHP evaluation and entropy weighting evaluation, respectively, and lastly, patient evaluation and expert evaluation were linearly integrated.

### 4.3. Model measurement and discussion

#### 4.3.1. Model measurement

Reliability and validity tests should be conducted prior to quantitative analysis to ensure the validity of the classification of potential variables in the model. The SPSS software is used for reliability tests. The study used Cronbach’s *α* to measure the reliability of the data, when the reliability coefficient is >0.8 indicates good reliability, 0.6–0.8 indicates acceptable reliability, when below 0.6 indicates poor reliability requires rearrangement of the variables in the RBI-SEM model. After testing, Cronbach’s *α* of *P_i_*, the minimum value, maximum value and average value are 0.807, 0.853, and 0.821 respectively; for *B_i_* are 0.702, 0.818, and 0.754 respectively; for *E_i_* are 0.816, 0.865, 0.852 respectively; for *S_i_* are 0.812, 0.868, and 0.789, respectively. Overall, the data are highly accurate, complete and reliable. The calculation results are shown in Table 3.

**Table 3:** Cronbach‘s alpha (*α*), composite reliability (CR), and average variance extracted (AVE).

	*P*	*B*	*S*	*E*	VRTL design
*α*	0.754	0.852	0.785	0.782	0.698
CR	0.877	0.768	0.809	0.783	0.736
AVE	0.708	0.458	0.523	0.484	0.487

Secondly, SPSS software is used to test the validity, which includes content validity, criterion validity and structure validity. Generally, content validity and construct validity are tested.

Moreover, because the good integrity of the statistical data, they are all evaluated after the VRTL test. The structure validity refers to the correspondence between a certain structure and the measured value reflected by the measurement results. The KMO and Bartlett sphericity of factor analysis method in SPSS software are commonly used for the structure validity test. When KMO is >0. 8, it is considered that the effect is good, 0.7–0.8 is fair enough, 0.6–0.7 is unacceptable, and the indicators should be re-selected when KMO is below 0.5. The test shows that the minimum KMO of *P* is 0.707, the maximum is 0.803, and the average is 0.718; *B* The minimum KMO of each explicit variable is 0.742, the maximum is 0.867, and the average value is 0.829; The minimum KMO of *E* explicit variable is 0.793, the maximum is 0.848, and the average value is 0.835; The minimum KMO of each explicit variable is 0.709, the maximum is 0.768, and the average is 0.793.The results are shown in Table 4.

**Table 4:** Fornell-Larcker discriminant validity (DV).

	*P*	*B*	*S*	*E*
*P*	0.708			
*B*	0.428	0.458		
*S*	0.405	0.485	0.523	
*E*	0.400	0.356	0.282	0.487
Square root of AVE	0.842	0.677	0.723	0.698

Table 5 shows that the goodness of fit index GFI, comparative fit index CFI. According to the table, the normative fit index NFI and value-added fit index IFI of the model are all >0.900, and the results are well matched. The root mean square of approximation error (RMSAE) equals 0.063, which is <0.080, and the results fit well. Chi square degree of freedom ratio *χ*^2^/df = 1.589 < 5. In conclusion, the overall convergence validity of VRTL model is good. The factor loads of the observed variables, which correspond to the four potential variables (physical perception, action perception, environmental perception and social perception) in the model, are all >0.5. It indicates that the corresponding observed variables are representative. The AVE of the mean variance variation corresponding to each dimension is >0.5000, and the combined reliability CR is >0.800, indicating that the convergence validity is ideal. Secondly, we verify the model discrimination validity. The obtained absolute values of the correlation coefficients of the five latent variables are <0.500 and are less than the square root of the corresponding AVE. The latent variables have a certain degree of discrimination, which means that the scale data have good discriminant validity.

**Table 5:** Fornell-Larcker discriminant validity (DV).

Fitting index	Reference standards	Output
GFI	>0.900	0.901
CFI	>0.900	0.957
NFI	>0.900	0.923
IFI	>0.900	0.956
RMSEA	<0.080	0.063
*χ*^2^/df	0 < *χ*^2^/df < 5	1.589

#### 4.3.2. Result of RBI-SEM model

**Figure 5:**
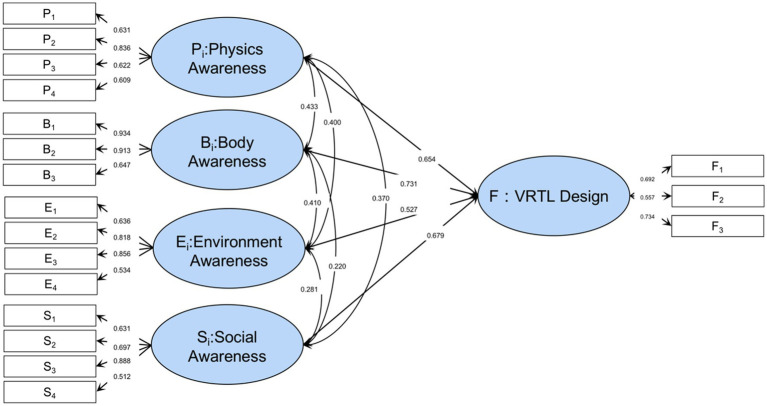
RBI-SEM model results.

In the previous step, we have finished measuring the RBI-SEM model and confirmed its strong convergence and discrimination validity. Table 6 displays the model standardization coefficient. The theoretical model’s latent variables are thought to positively affect the VRTL design, indicating that design optimization in these four areas can also benefit the VRTL design. The final RBI-SEM model results are shown in Figure 5. The Standardized path coefficients of the other measured variables can be seen in Table 7.

**Table 6:** Preliminary results of structural equation modeling analysis.

Path	Standardized coefficients	SE	CR	Value of *p*
*P* → *F*	0.217	0.044	4.236	***
*B* → *F*	0.130	0.047	3.743	*
*E* → *F*	0.243	0.045	3.609	***
*S* → *F*	0.122	0.043	2.551	***

*means 0.01 < *p* < 0.05; ***means *p* < 0.001.

**Table 7:** RBI-SEM design element weighting statistical table based on multiple weighting methods.

Latent variable	Path coefficient of latent variable	Measurable variable	Estimate	SEM based weight	AHP weight	Entropy weight	Comprehensive weight
*P_i_*	0.217	*P*_1_ Light environment	0.836	0.0826	0.5901	0.574	0.664
		*P*_2_ Acoustic environment	0.622	0.0615	0.510	0.159	0.396
		*P*_3_ Color suitability	0.609	0.0602	0.556	0.199	0.437
		*P*_4_ Scene texture tactile perception	0.613	0.0606	0.495	0.068	0.342
*B_i_*	0.130	*B*_1_ Interactive scenes	0.934	0.0923	0.581	0.333	0.549
		*B*_2_ Boundary space	0.913	0.0902	0.561	0.297	0.519
		*B*_3_ Travel speed	0.647	0.0639	0.557	0.164	0.424
*E_i_*	0.243	*E*_1_ Scene scale	0.818	0.0808	0.522	0.078	0.381
		*E*_2_ Scene artistry	0.856	0.0846	0.511	0.136	0.408
		*E*_3_ Vegetation diversity	0.534	0.0528	0.671	0.558	0.667
		*E*_4_ Water landscape	0.636	0.0628	0.610	0.228	0.482
*S_i_*	0.122	*S*_1_ Nostalgic elements of memories	0.636	0.0628	0.517	0.083	0.363
		*S*_2_ Identification system	0.697	0.0689	0.509	0.267	0.457
		*S*_3_ Accessible roaming space	0.887	0.0877	0.429	0.496	0.551
		*S*_4_ Service elements	0.512	0.0506	0.437	0.154	0.346

#### 4.3.3. Integrated weighting calculation

SEM calculation shows that each potential variable has a positive effect on VRTL design satisfaction. The next step is to rank the indicators of each measurable variable. However, the action path coefficient cannot directly reflect the weight of measurable variables or guide design practice. By constructing a normalized algorithm for path coefficients, combining traditional subjective and objective comprehensive weighting methods, and fully balancing subjective and objective weights, a statistical table of RBI-SEM design elements based on multiple weighting methods is obtained, as shown in Table 7. where, based on the standardized path coefficient in the SEM model, the weight index of each measurable variable based on SEM is obtained by using Equations (2)-(5), as shown in columns 4 and 5 of Table 7; Based on the subjective weighting method AHP, the subjective weight index of each measurable variable is gained by using Equations (6)-(9), as shown in the column 6 of Table 7; Based on entropy weight method, the objective weight are gotten by using Equations (10)-(14), as shown in the column 7 of Table 7; Finally, according to Equation (15), the weights are linearly combined to obtain a rational importance ranking of design indicators, which can be used to guide the VRTL design, as shown in the column 8 of Table 7.

#### 4.3.4. Pre-test evaluation results

VRTL can address the landscape requirements of older adults with depressive tendencies and provide positive psychological and physical interventions, while also being able to reduce the medical burden and provide intelligent strategies for a variety of chronic conditions (28), and its currently an effective aid in preventing and combating depression (10, 29). A summary of the VRTL design elements recommended for use in PSD is shown in Table 8.

**Table 8:** Summary of the design indicators of each pathway.

Latent variable name	Core design indicators	Optional design indicators
*P_i_*	*P*_1_ Light environment	*P*_3_ Color suitability
*B_i_*	*B*_1_ Interactive landscape	*B*_2_ Boundary space
*E_i_*	*E*_3_ Vegetation diversity	*E*_4_ Water landscape
*S_i_*	*S*_3_ Accessible roaming space	*S*_2_ Identification system

Our results concluded that RBI-SEM verifies *P_i_* physical awareness, *B_i_* body awareness, *E_i_* environmental awareness, and *S_i_* social awareness significantly correlates with VRTL design (*P* >> *F* 0.217; *B* >> *F* 0.130; *E* >> *F* 0.243; *S* >> *F* 0.122).For the latent variables the importance ranking is *E_i_* (*p* < 0.001), *P_i_* (*p* < 0.001), *S_i_* (*p* < 0.001), *B_i_* (*p* < 0.05). It indicates that the environmental ambience and landscape in the virtual environment are most essential, and it is the most notable difference between VRTL and physical treatment approaches. In the construction of VRTL, from the perspective of comprehensive consideration, more emphasis should be placed on highly immersive landscape display methods such as panoramic scenes in VR to make full use of the sensory stimulation brought about by virtual technology, thus maximizing the effectiveness of VR-based rehabilitation elements (e.g., the drinking terrace, sculpture, and flower bed in the VRTL experiment). Secondly, patients also consider the physical perception element relatively important after the experience, which indicates that it is necessary to provide basic behavioral simulation in VR, and that multi-sensory interaction is more conducive to immersive VRTL experiences.

In the ranking of latent variables, importance can be roughly ranked according to the magnitude of the standardized path coefficients (e.g., *B_i_* body awareness is already lower in correlation coefficients), while secondary indicators continue to proceed with AHP and entropy evaluation according to the RBI-SEM model. In environmental awareness (0.243). the *B* comprehensive evaluation weight (0.667) is the highest among the measured variables, indicating that the vegetation scene in VRTL can maximize user satisfaction, and the vegetation in VR is not only an important carrier of color but also the main form of space, and the overall style of natural vegetation is recognized by patients, while the AHP weight (0.671) and entropy weighting method (0.558) were both higher and consistent. The overall evaluation weighting of *B* (0.482) ranked second, indicating that a highly restored hydrological landscape can provide a better healing atmosphere, and combined with the vegetation landscape, most patients in this study tended to use the VR system to experience the natural landscape. In contrast, patients’ demand for evaluation of human landscape and landscape artistry was weaker, which may be due to the poor reproduction of various details of human art by existing VR devices and modeling methods, resulting in the inability of this element to attract the attention of the subjects.

Moreover, the physical awareness (0.217) was relatively important, but combined with its AHP and entropy weight evaluation, the importance was lower than *E_i_*. The comprehensive importance order of the measurable variables was light environment (0.664) > color suitability (0.437) > sound environment (0.396) > scene texture band option (0.342). The evaluation results show that the visual environment and visual color in VRTL are the priority of users’ interests, and the design of the VRTL test software, which focuses on outdoor illumination and natural color restoration, is accepted by PSD patients. However, the evaluation of the auditory environment was relatively low, which may be due to the generally older age of the sample in this study, the relatively poor auditory ability, and the lack of auditory equipment for the VR environment, so the auditory reproduction was poor and did not provide satisfactory outcomes.

Social awareness (0.122) was significantly associated with VRTL design, but its path coefficient was relatively low. The order of importance of its measurable variables is: accessible roaming space (0.551) > identification system (0.457) > nominal elements of memory (0.363) > service elements (0.346), indicating that in the construction of social perception of VRTL, the spatial boundary restrictions and scale space changes should be fully utilized, which can realize the multi-level display of space, create easy to perceive and roam spatial forms that enhances the interaction and immersion of older adult people with depressive tendencies.

Lastly, body awareness (0.130) was significantly correlated with VRTL, but the path and correlation coefficients were both low, and the importance ranking of the measurable variables was interactive scenes (0.549) > boundary space (0.519) > travel speed (0.414). The SEM results showed low importance. However, the combined evaluation weight of interactive landscape in the group (AHP: 0.58; entropy weight: 0.33; combined: 0.55) is the highest among the potential variables, indicating that the interactive landscape in VRTL is considered to be able to satisfy the VRTL treatment to the maximum extent in the expert evaluation. In the subsequent design, the tactile feedback of natural landscape and the addition of treatment-related flora and fauna images can be designed to enrich the means of interaction in VRTL.

### 4.4. Post-test

#### 4.4.1. Effect of VRTL on physiology of subjects and correlation analysis

The physiological data before VRTL treatment is used as the physiological index in the hospital environment, while the physiological data after VRTL treatment serves as the post-test data, by evaluating the differences between the changes in patients’ physiological response values in the hospital environment and after VRTL treatment.

**Figure 6:**
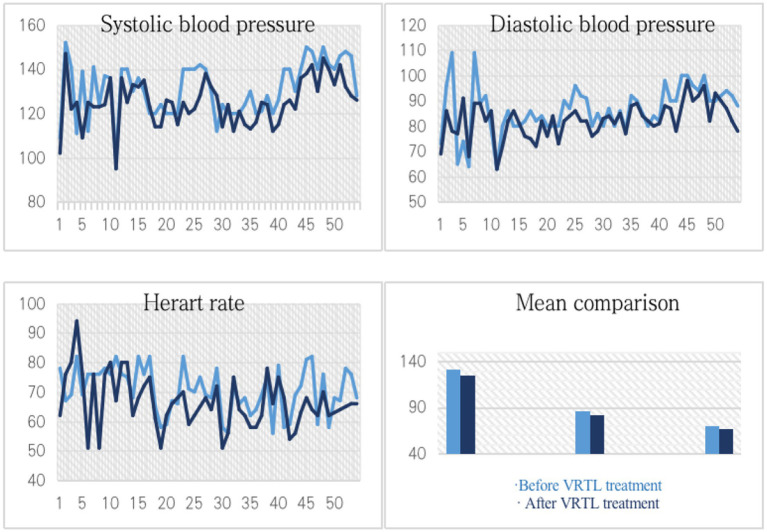
Changes of blood pressure, heart rate and their mean values before and after VRTL treatment.

Before and after participating in the virtual rehabilitation environment experience, older adults with depression tendencies have their diastolic blood pressure, systolic blood pressure, and heart rates compared. The outcomes are displayed in Figure 6. The extent and frequency of their drop and growth are similar before and after the virtual rehabilitation landscape experience, yet there is a shift in the total statistics. Systolic and diastolic blood pressure decreases are larger than increases with a similar frequency of occurrence, showing a general downward tendency. The heart rate of patients with depression tendencies has been greatly decreased through the virtual rehabilitation landscape experience, as shown by the fact that the decrease in heart rate is significantly greater than the increase in heart rate in degree and frequency.

It is possible to find whether there is a significant difference between the mean value of pre-test data and the test value of post-test data based on the *T*-test analysis results of paired samples of pre-test and post-test data provided in Table 9. The results show that the statistical *T* values are all normal distribution, and *p*-values are all <0.01. Therefore, the mean blood pressure and heart rate after the assessment are significantly different from those before the assessment. Combined with the changing trend, it can be considered that the virtual rehabilitation landscape experience has a positive impact on the physiological health of older adults with depression tendencies and can effectively reduce human blood pressure and heart rate.

**Table 9:** *T*-test of paired samples for pre-test and post-test of blood pressure and heart rate.

Physiological data	Paired difference					*T*	df	Value of *p*
	M	STD	Standard error of mean	Confidence interval				
				lower limit	Upper limit			
Systolic pressure	6.09259	9.12237	1.24140	3.60266	8.58252	4.908	53	0.0001
Diastolic pressure	4.11111	7.37581	1.00372	2.09790	6.12432	4.096	53	0.0001
Herat rate	4.11111	8.37261	1.13937	1.82583	6.39639	3.608	53	0.001

#### 4.4.2. Relevant factors affecting physiological rehabilitation effect

When compared to the pre-test, the systolic and diastolic blood pressure of the older adult with depression tendencies reduce to a certain level after experiencing VRTL, according to the study. It suggests that VRTL positively impacts older adults’ physical health. The post-test blood pressure and heart rate data of older persons with depressive tendencies are evaluated by one-way ANOVA with gender and age to determine the relationship between the fundamental characteristics of the comparative sample and the physiological data. According to the results of the correlation analysis in Table 10, *p*-value > 0.01, it can be considered that there is no significant difference in the changes in blood pressure and heart rate among the subjects of different ages and genders.

**Table 10:** Correlation between blood pressure and heart rate in users of different genders and ages.

	Sex		Age	
	*F*	Value of *p*	*F*	Value of *p*
Systolic pressure	4.877	0.32	2.183	0.102
Diastolic pressure	2.209	0.143	1.160	0.334
Herat rate	0.931	0.339	2.331	0.085

By comparing the correlation between physiological data and the virtual rehabilitation landscape factor evaluation system, the bivariate equation of simple regression and the multivariate linear regression equation are performed for the reduction of post-measured diastolic blood pressure, systolic blood pressure and heart rate with the results shown in Table 11. It can be seen from the coefficient of the equation that scene scale and scene art in VRTL have a positive impact on systolic blood pressure, while scene art benefits diastolic blood pressure, and the reduction of the heart rate of vegetation diversity and water landscape also has a positive impact.

The data shows that the positive effect of VRTL on the blood pressure of older adults with depression tendencies is mainly due to the setting of TL. Also, the immersive qualities and other features provided to users by VR technology, such as interactive richness and landscape aesthetics, form a notable contrast with the real landscape space. The former can make PSD patients who experience virtual scenes more relaxed physically and mentally. Their heart rate is also positively affected by the virtual landscape elements, especially the water landscape, which shows that a reasonable hydrological landscape layout can help depressed older adults release pressure and calm their emotions.

**Table 11:** Impact of design elements in VRTL on blood pressure and heart rate.

	Interactive landscape	Scene artistry	Vegetation diversity	Water landscape	*R* ^2^	*F*	Value of *p*
Systolic pressure	–	0.012^*^	–	–	0.588	0.571	0.019
Diastolic pressure	0.471^*^	0.171^*^	–	–	0.661	0.781	0.018
Herat rate	–	–	0.247^*^	0.227^*^	0.672	0.818	0.016

* Significant correlation at 0.05 level (both sides).

### 4.5. Strengths and limitations

In our research, the RBI-SEM evaluation model was established and quantitatively should confirm the significant correlation of *P, B, E, S* on VRTL in RBI theory, which can guide the design practice in more detail than the conventional structural equation and regression analysis studies. And in the post-test, it was concluded that the changes in physiological indicators were significant, and the VRTL utility was empirically analyzed by regression analysis that measured variables such as Interactive landscape in the study were significantly correlated with the decrease in blood pressure and heart rate of patients.

However, the questionnaire sample size provided by 88 patients was relatively insufficient, which led to a one-sidedness of the SEM results, and RBI-PRS scale was difficult to understand for order adult PSD patients. Additionally, in the physiological index measurement (before VRTL experience), patients were not given enough rest time, which may have some influence on the correlation test.

## 5. Discussion

Our study quantitatively confirmed that *P, B, E, S* are significantly correlated with VRTL treatment utility, which has a positive guiding effect on the promotion of patient subjective well-being in VRTL, and more previous qualitative studies have similar findings (2, 6, 18); on the other hand, the comprehensive evaluation method proposed based on the RBI-SEM model can help designers better decompose design tasks from multiple perspectives to guide design practice in a clear sequence.

According to the results of the pre-test assessment, environment awareness is important, among which scene artistry and scene scale are not surprising, as our aesthetic judgments are always evolving due to our biophilia characteristics (2, 3), and the visual strengthening in the VR environment makes these two elements, which are mainly visual perception, more easily noticeable (9). Of particular interest are vegetation diversity and water landscape, both of which emphasize specific natural landscape requirements and have a high level of importance. In many scholars’ researches, such as Chen and Sidik (25, 30), it was emphasized that the construction of humanistic landscapes (e.g., parks, libraries, rooftops, observation decks), which are public places with interpersonal interaction implication, has an important role in citizens’ subjective well-being (31), whereas natural landscapes (e.g., vegetation diversity,light simulation, water landscapes, etc.) were evaluated significantly higher than humanistic landscapes in both experiments. This may be due to the common negativity of PSD patients in interpersonal communication (32), while previous conventional psychotherapy for PSD would guide patients to participate in socialization and establish dialogues to relieve psychological stress (7, 10, 33, 34), compared to the experiments in VRTL which did not provide sufficient guidance and had potential for upgrading.

The insight that VR technology is more adept at visual and auditory simulations arose several times (35), which resulted in physical awareness and social awareness pathway in light environment, color suitability, identification system, and other visual-mediated measurement variables tend to obtain better assessment results. Moreover, social awareness was significantly correlated with VRTL in the pre-test, and in Brakel’s study social awareness in the VR environment was found to be connected with the self-perception of existence and social behavioral support (e.g., hugging, touching one’s shoulder, patting one’s back) (36), and the construction of VR communities and platforms is thought to enhance social awareness as a manifestation of communicative tendencies, which is the same as the need to strengthen the identification system as suggested in the results of the pre-test experiment (37). VRTL therapy requires patients to be able to clearly perceive their social presence and thus restore interpersonal communication, which can refer to the above-mentioned studies to establish online conversations, sports activities. Simultaneously, the function of verbal interactivity may accompany by security issues, aggressive and harassing behavior in VR environments, to be noticed by developers (38).

Currently, VRTL is relatively rare in clinical treatment, and the lack of unified design standards and ideology limits its promotion, while recently researches of VRTL have been more in exploring the latent relationship between VR technology and treatment outcomes (39, 40). The RBI-SEM evaluation established in this study can be well used as an analytical tool to decompose patient needs from four dimensions (RBI) with a designer’s perspective. Taking the VRTL design of PSD as an example, by enhancing the social platform, promoting patients’ social awareness, and providing a better healing atmosphere through natural landscape and lighting, these elements can serve as a good healing guide for PSD patients.

## 6. Conclusion and future research

With the use of VR technology, VRTL is a dependable instrument that gives patients appropriate exercise opportunities and aids in the treatment of mental illnesses. This paper chooses PSD as the design practice direction and investigates how to design, evaluate, and verify VRTL for older adults with PSD in a hospital setting. This is done in order to reasonably integrate VR technology into the treatment process and build a VRTL design method centered on patient experience. Two major innovations include: (1) A VRTL evaluation model based on SEM, AHP, and the entropy weight method is built on the basis of user experience evaluation research. In contrast to traditional evaluation methods, the evaluation model is checked before being utilized to gather scoring information on the Likert scale for a comprehensive evaluation that can accurately reflect the patient’s opinions. (2) The effectiveness of the design process and the actual effectiveness of VRTL for PSD are verified through the measurement of physiological indicators. The scoring data shows that visual design elements like hydrological environment (0.227) and vegetation landscape (0.247) are significantly related to the decline in heart rate while scene scale (0.442) and scene art (0.121) are strongly related to the decline in blood pressure (diastolic pressure), which supports the efficacy of the VRTL design method.

Nevertheless, this study uses multiple assessment methods to evaluate the construction of the VRTL design process. However, because the relative sample size of the indicator data obtained using the Likter scale is insufficient and limited to the scoring data and the same time period, the research’s universality needs to be enhanced. Nevertheless, this article verifies the curability of VRTL in the ward unit through the follow-up physiological index test. Therefore, the inclusion of quantitative data collection methods such as eye movement and anthropometry will be more conducive to the evaluation of objectivity of future research on VR technology evaluation methods. And plus, VRTL, as a successful method to replace the actual landscape, can provide relaxation and a certain amount of exercise in the treatment. However, in the application, patients have not paid attention to the interaction approach based on other senses. So, the realization of multimodal interaction in VRTL requires targeted hardware development for the senses other than vision to improve the immersive experience.

In general, the construction of the comprehensive evaluation system of VRTL based on RBI-SEM can be seen as a reasonable attempt to integrate VR technology into the traditional medical system, and show its efficacy in the practical application of PSD treatment, laying a foundation for the future large-scale clinical application. It is believed that in the future, VRTL can be organically integrated into more medical procedures according to the design process and become a part of common medical means.

## Data availability statement

The raw data supporting the conclusions of this article will be made available by the authors, without undue reservation.

## Ethics statement

The studies involving human participants were reviewed and approved by Ethics Committee of Wuhan Jinyintan Hospital. Written informed consent for participation was not required for this study in accordance with the national legislation and the institutional requirements.

## Author contributions

YL: study concept and design, image preprocessing, analysis and interpretation of data, and drafting of the manuscript. QZ: acquisition of data. QZ and XF: critical revision of the manuscript for important content. All authors contributed to the article and approved the submitted version.

## Conflict of interest

The authors declare that the research was conducted in the absence of any commercial or financial relationships that could be construed as a potential conflict of interest.

## Publisher’s note

All claims expressed in this article are solely those of the authors and do not necessarily represent those of their affiliated organizations, or those of the publisher, the editors and the reviewers. Any product that may be evaluated in this article, or claim that may be made by its manufacturer, is not guaranteed or endorsed by the publisher.
